# Tractography for Subcortical Resection of Gliomas Is Highly Accurate for Motor and Language Function: ioMRI-Based Elastic Fusion Disproves the Severity of Brain Shift

**DOI:** 10.3390/cancers13081787

**Published:** 2021-04-09

**Authors:** Sebastian Ille, Maximilian Schwendner, Wei Zhang, Axel Schroeder, Bernhard Meyer, Sandro M. Krieg

**Affiliations:** 1 Department of Neurosurgery, School of Medicine, Klinikum rechts der Isar, Technical University of Munich, 81675 Munich, Germany; Sebastian.Ille@tum.de (S.I.); Maximilian.Schwendner@tum.de (M.S.); wei.zhang@tum.de (W.Z.); Axel.Schroeder@tum.de (A.S.); Bernhard.Meyer@tum.de (B.M.); 2 TUM Neuroimaging Center, School of Medicine, Klinikum rechts der Isar, Technical University of Munich, 81675 Munich, Germany

**Keywords:** glioma, intraoperative MRI, intraoperative neuromonitoring, paresis, tractography

## Abstract

**Simple Summary:**

Tractography enables the visualization of eloquent white matter pathways. Inaccuracies due to brain shift might occur intraoperatively. The aim of this study was to evaluate the impact of intraoperative magnetic resonance imaging (MRI)-based elastic fusion on preoperative tractography for subcortical resection of gliomas. We confirmed the high accuracy of tractography during the whole course of surgery.

**Abstract:**

When using preoperative tractography intraoperatively, inaccuracies due to brain shift might occur. Intraoperative tractography is rarely performed. Elastic fusion (EF) is a tool developed to compensate for brain shift, gravity, and tissue resection based on intraoperative images. Our hypothesis was that preoperative tractography is accurate and adjustments of tractography by intraoperative magnetic resonance imaging (ioMRI)-based EF (IBEF) compensate for brain shift. Between February 2018 and June 2019, 78 patients underwent eloquent (46 motor, 32 language) glioma resection in our department using intraoperative MRI. Mean distances between the resection cavity and tractography were analyzed and correlated with clinical outcomes. The mean ± standard deviation (range) distance after the application of IBEF was 5.0 ± 2.9 mm (0–10 mm) in patients without surgery-related motor deficits compared with 1.1 ± 1.6 mm (0–5 mm) in patients who showed new permanent surgery-related motor deficits postoperatively (*p* < 0.001). For language, the distance was 0.7 ± 1.2 mm (0–2 mm) in patients with new permanent deficits compared with 3.1 ± 4.5 mm (0–14 mm) in patients without new permanent surgery-related language deficits (*p* = 0.541). Preoperative tractography corrected by IBEF for subcortical resection of gliomas is highly accurate. However, at least for such subcortical anatomy, the severity of brain shift was considerably overestimated in the past.

## 1. Introduction

For the resection of eloquent brain tumors, a maximum extent of resection (EOR) should be achieved while functionality must be preserved to provide optimal oncological treatment [1,2,3,4,5]. 

Tractography enables the visualization of eloquent subcortical pathways prior to surgery, helping to identify subcortical structures, such as the corticospinal tract (CST) or arcuate fascicle, in relation to the tumor. Compared with the gold standard of intraoperative direct subcortical stimulation, tractography shows good concordance [6,7,8,9,10]. 

During surgery, opening of the dura, loss of corticospinal fluid, and tumor resection cause an increasing brain shift. This might pose the risk of increasing the inaccuracy of tractography, especially at the end of tumor resection, where tractography might be of most importance.

Centers routinely use intraoperative magnetic resonance imaging (ioMRI). Recent studies have shown that the application of ioMRI leads to improvements in EOR, overall survival, and the patients’ quality of life [11,12,13,14]. Similarly, ioMRI supplies data regarding the current status of the tumor resection, showing the brain with open dura and the current extent of any brain shift. For further resection after ioMRI, scans can serve as a neuronavigation equal to the preoperative MRI scans, providing more accurate data [15]. However, intraoperative diffusion images are rarely performed due to technical limitations of the scanner, coils, head clamp, or due to the duration of the ioMRI scan. 

The pure rigid projection of preoperative diffusion tensor imaging (DTI) data, such as fiber tractography (FT) on anatomic ioMRI scans, can cause considerable spatial inaccuracies [16,17]. Therefore, algorithms of elastic fusion (EF) have been developed. EF reduces geometric image distortion between preoperative MRI scans and ioMRI scans using algorithms of distortion correction (DC) [16]. This enables the fusion of MRI sequences at different timepoints and can be adjusted even for severe anatomical changes such as tumor resection and intraoperative brain shift. Tractography after ioMRI-based EF (IBEF) showed high accuracy and high reliability in comparison with intraoperative direct subcortical stimulation with regards to clinical outcomes in an analysis of eleven patients [18]. ioMRI-based EF (IBEF) can therefore be used to adjust preoperative FT to anatomical ioMRI scans and thereby make the extensive preoperative workup and planning available for intraoperative use after ioMRI. Similarly, IBEF enables the comparison of preoperative and intraoperative imaging of the full extent of brain shift and the impact of brain shift on tractography.

The aim of this study was to evaluate whether the fusion of preoperative tractography and intraoperative anatomical MRI scans provides accurate data correlating with the patients’ postoperative clinical outcomes, and how tractography corrected by IBEF can compensate for intraoperative brain shift to provide improved risk assessment regarding further resection after ioMRI.

## 2. Materials and Methods

### 2.1. Ethics

The study was approved by the local ethics board (registration number: 336/17, 192/18). We performed the study in accordance with the Declaration of Helsinki. All patients included in this study provided written informed consent.

### 2.2. Study Protocol

The aim of this study was to evaluate the impact of intraoperative brain shift during glioma resection on the accuracy of preoperative motor and language tractography. Therefore, we correlated the patients’ functional outcomes with the distance between the resection cavity on ioMRI and the preoperatively performed tractography. Analysis was performed for tractography with and without correction by IBEF. We prospectively included patients with brain lesions scheduled for microsurgical resection. Exclusion criteria were age < 18 years as well as general MRI exclusion criteria such as cochlear implants or cardiac pacemakers.

For the exact seeding of function-based DTI FT, we used navigated transcranial magnetic stimulation (nTMS) to identify language and motor eloquent brain areas. Patients with eloquent fiber tracts regarding tumor location and planned surgical approach for resection identified on preoperative MRI were included in this study. Microsurgical resection of the tumor was performed using neuronavigation as well as continuous motor evoked potential (MEP) monitoring in all cases. Intraoperative neuromonitoring including direct cortical stimulation and subcortical stimulation was additionally performed in selected cases.

After completing the resection, an ioMRI scan was acquired. IBEF was subsequently performed based on the ioMRI scans. Based on the findings from the ioMRI scan, resection was either continued or closure of the field was started. Motor and language status were examined 5 days as well as 3 months postoperatively.

### 2.3. Diffusion Tensor Imaging Fiber Tracking of Eloquent Fiber Tracts

All patients obtained a structural MRI scan (3T MR scanner Achieva 3T, Philips Medical System, Netherlands B.V.) according to the standard MRI protocol, including a 3D fluid-attenuated inversion-recovery (FLAIR) sequence, a 3D gradient echo sequence with intravenous contrast administration (T1 + CA), and DTI sequences with 32 orthogonal sequences prior to surgery. For the visualization of fiber tracts representing motor function, we set the MEP-positive cortical sites as determined by preoperative nTMS mapping, as well as the ipsilateral brain stem, as regions of interest (ROIs) for DTI FT.

For the tractography of language eloquent subcortical pathways, positive nTMS language cortical sites were set as ROIs. Tractography was performed using a standard deterministic algorithm with a fiber assignment by continuous tracking (iPlanNet Cranial 3.0.1, Brainlab AG, Munich, Germany). Therefore, all fiber tracts related to language function within the fractional anisotropy threshold were included. The focus was on the arcuate fascicle, the inferior fronto-occipital fascicle, the superior and inferior longitudinal fascicles, the frontal aslant tract, and the uncinate fascicle.

### 2.4. Intraoperative MRI

At our institution, a two-room ioMRI setup was used (3T MR scanner Ingenia, Philips Medical System, Netherlands B.V.). For the ioMRI scan, an MRI-compatible head clamp, including an 8-channel coil array (Noras MRI products, Hoechberg, Germany) was used. Prior to the ioMRI scan, the resection cavity was filled with Ringer’s solution and closed with Surgicel (Ethicon, Somerville, NJ, USA) and two-stich sutures. The situs was then covered with multiple sterile layers. Needles for intraoperative neuromonitoring were removed for safety and image quality reasons. After completing the ioMRI checklist, the patient was transferred to the ioMRI under constant general anesthesia.

### 2.5. ioMRI-Based Elastic Fusion

During the ioMRI scan, the anatomical 3D T1 sequence was acquired first. It was then used for the fusion of preoperative and intraoperative images. Next, the IBEF algorithm was applied, enabling calculation of tissue deformation based on the comparison of preoperative and intraoperative images including finite element analysis and gravity. 

**Figure 1:**
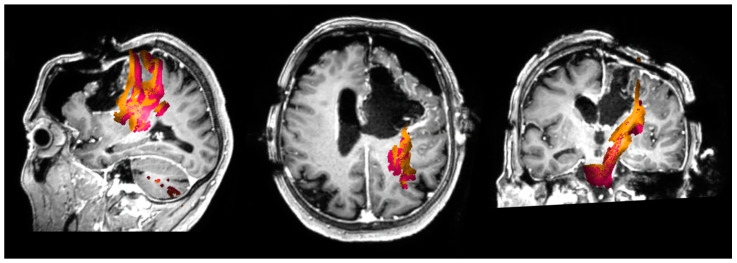
Navigated transcranial magnetic stimulation-based (nTMS) motor tractography corrected by intraoperative magnetic resonance imaging-based elastic fusion (IBEF)in an intraoperative magnetic resonance imaging (ioMRI)scan. This figure shows motor fiber tracts based on navigated transcranial magnetic stimulation (nTMS) in an intraoperative magnetic resonance imaging (ioMRI) scan in an exemplary patient case. The preoperative motor fiber tracts are shown in orange. The fiber tracts corrected by ioMRI-based elastic fusion (IBEF) are shown in pink.

**Figure 2:**
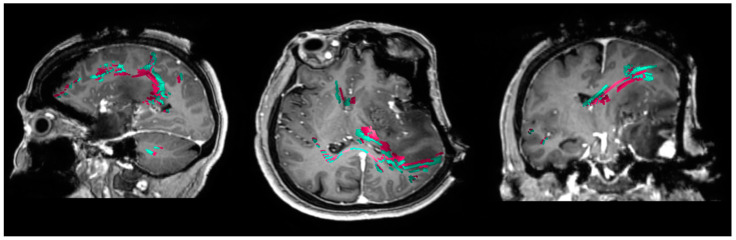
nTMS-based language tractography corrected by IBEF in an ioMRI scan. This figure shows motor fiber tracts based on navigated transcranial magnetic stimulation (nTMS) in an intraoperative magnetic resonance imaging (ioMRI) scan in an exemplary patient case. The preoperative motor fiber tracts are shown in pink. The fiber tracts corrected by ioMRI-based elastic fusion (IBEF) are shown in cyan.

The IBEF algorithm enables adjustments of preoperatively determined objects including DTI-based fiber tracts according to the current intraoperative anatomic conditions specified by ioMRI. After manual verification of the calculated EF, preoperatively determined objects including tractography were fused with the ioMRI and used for further resection (Brainlab Elements^®^, Brainlab AG, Munich, Germany) (Figure 1 and Figure 2).

### 2.6. Data Analysis

Regarding the extent of resection, the commonly used threshold of 5% of postoperative tumor residue was used to differentiate between gross total resection (GTR) and subtotal resection (STR) [19,20]. Clinical examination was performed preoperatively, postoperatively, and at the 3 month follow-up. Motor status was determined in accordance with the British Medical Research Council (BMRC) scale. Language function was documented using our standard grading of aphasia adapted from the Aachen Aphasia Test (AAT) [21,22]. The minimum distance between the resection cavity in the ioMRI and eloquent fiber tracts corrected with IBEF was analyzed (Figure 1 and Figure 2). Therefore, we chose sequences that are essential for the evaluation of a residual tumor (FLAIR for low-grade gliomas; T1 + CA for high-grade gliomas).

Statistical analyses were performed using Prism (version 8.4.1; GraphPad Software, La Jolla, CA, USA). Descriptive statistics were calculated for patient- and tumor-related characteristics as well as for distances between fiber tracts and the resection cavity. Patient cases without surgery-related deficits were compared with patients with surgery-related deficits. Furthermore, the incidence of surgery-related deficits was analyzed in relation to the distance between the resection cavity and the fiber tracts. Therefore, Mann–Whitney U tests for unpaired samples with a level of significance set at *p* < 0.05 were performed. For the comparison between measurements with and without the use of IBEF as well as measurements preoperatively, Wilcoxon tests for paired samples with a level of significance set at *p* < 0.05 were applied.

## 3. Results

### 3.1. Patient Characteristics

We analyzed 78 cases of patients harboring eloquent gliomas with preoperative nTMS fiber tracking and intraoperative MRI. In 46 cases, preoperative nTMS motor fiber tracking was performed. The site of the tumor was left-hemispheric in 24 cases and right-hemispheric in 22 cases. 

We accordingly analyzed 32 cases of left-hemispheric language eloquent brain tumors with preoperative nTMS language fiber tracking and intraoperative MRI. Resection was performed as awake surgery in 12 patients.

Detailed clinical information of the patient cases with motor fiber tracking, including details on the motor status at different time points, is shown in Supplementary Table S1. Comparing the preoperative motor status to the motor status 5 days postoperatively, 19 patients declined in motor function at least one grade on the BMRC scale, whereas one patient improved. In total, 14 patients were lost to the follow-up examination three months postoperatively, among which six patients showed no surgery-related decrease in postoperative motor status.

For cases with language fiber tracking, detailed clinical information, including details on the language status at different time points, is shown in Supplementary Table S2. When comparing the preoperative and postoperative language status according to the modified AAT, ten patients declined in language function. 

Comparing the postoperative language status to the language status at the 3 month follow-up examinations, there were eight cases of improvement in language status, and one patient was lost to follow-up at the 3 month follow-up examination.

### 3.2. ioMRI Data

Performing both the intraoperative MRI and the elastic fusion of the preoperative fiber tracking for motor and language function was feasible in all of the enrolled patients. Regarding all 46 cases with motor eloquent fiber tracts, GTR on the ioMRI scan was achieved in 39 cases (84.8%) (Supplementary Table S1). Further resection was performed after the intraoperative MRI examination in 13 cases (28.3%). The mean overall prolongation of surgery duration due to ioMRI was 65 ± 20 min (30–105 min). Among 32 cases with language fiber tracking and intraoperative MRI examination, gross total resection on the ioMRI scan was achieved in 20 cases (62.5%) (Supplementary Table S2). Further resection was performed in eight cases (25.0%) after the ioMRI scan. The intraoperative MRI examination took 64 ± 18 min (35–100 min) on average. 

### 3.3. Elastic Fusion and Clinical Outcome

Regarding all patient cases with tractography of motor fibers, the mean distance between the resection cavity and the fiber tracts was 3.6 ± 3.2 mm (0–10 mm) after IBEF compared with 2.7 ± 3.0 mm (0–9 mm) before IBEF and 3.2 ± 3.5 mm (0–15) for the preoperative measurement (Table 1). The mean distance between the resection cavity and the fiber tracts was significantly higher for IBEF compared with cases without IBEF (*p* = 0.001).

**Figure 3:**
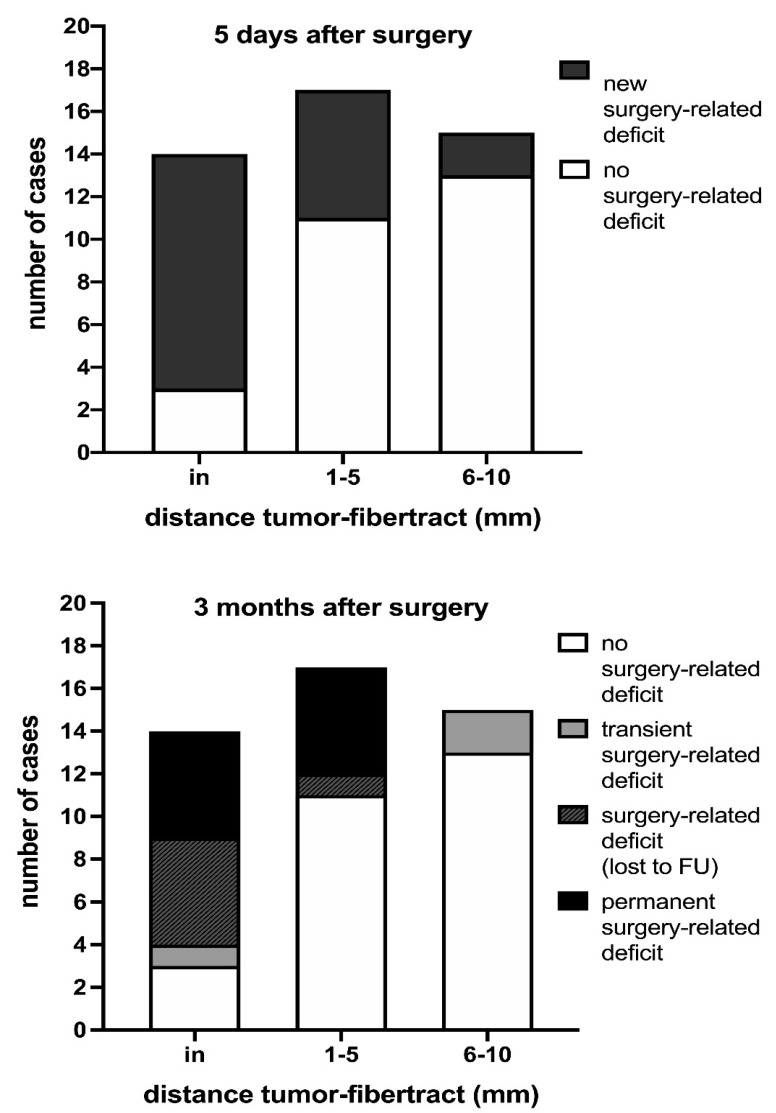
Prevalence of surgery-related deficits in relation to the distance between motor eloquent fiber tracts to the intraoperative MRI resection cavity. This figure outlines the surgery-related decrease in motor function in relation to the distance between the resection cavity and motor eloquent fiber tracts. Surgery-related motor deficits are significantly lower for distances of 1–5 mm and 6–10 mm (*p* = 0.029 and *p* = 0.001, respectively) compared with patients with motor eloquent fiber tracts located in the resection cavity. Similar results were found for permanent surgery-related motor deficits, including patients lost to follow-up, for distances of 1–5 mm and 6–10 mm (*p* = 0.073 and *p* < 0.001, respectively).

The distance between the resection cavity and motor eloquent fiber tracts measured after IBEF was 1.1 ± 1.6 mm (0–5 mm) for cases with permanent surgery-related deficits, including patients with postoperative deficits lost to follow-up at the examination 3 months postoperatively compared with 5.0 ± 2.9 mm (0–10 mm) for cases with transient or no postoperative deficits and therefore without deficits at 3 months’ follow-up (*p* < 0.01) (Figure 3). No deficits occurred for a distance > 5 mm, with a positive predictive value of 0.533 for permanent deficits for a distance < 5 mm.

**Figure 4:**
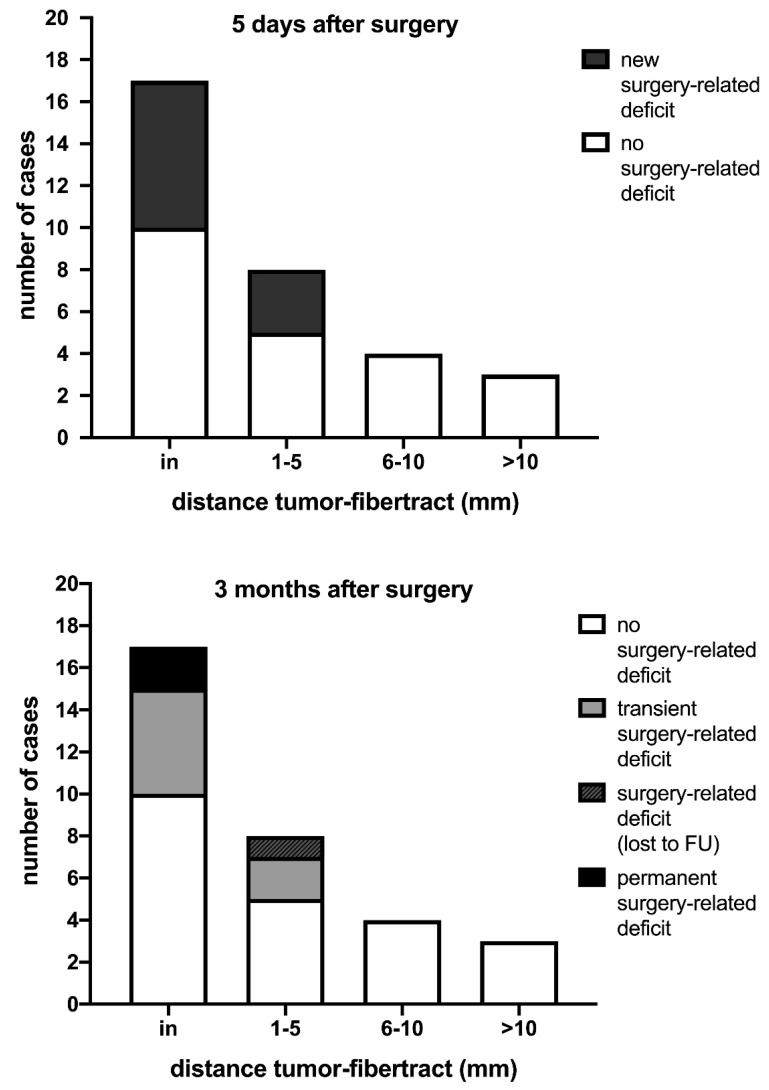
Prevalence of surgery-related deficits in relation to the distance between language eloquent fiber tracts to the intraoperative MRI resection cavity. The surgery-related decrease in language function in relation to the distance between the resection cavity and language eloquent fiber tracts is described in these figures. For a distance greater than 5 mm, no surgery-related deficit was observed. Surgery-related language deficits 5 days postoperatively were lower for distances of 6–10 mm and 10–15 mm (*p* = 0.255 and *p* = 0.521, respectively) compared with patients with language eloquent fiber tracts located in the resection cavity. Similar results were found for permanent surgery-related language deficits, including patients lost to follow-up, for distances of 5–10 mm and 10–15 mm (*p* > 0.999 each).

For patients with tractography of language function, the average distance between the resection cavity and language fiber tracts was 2.9 ± 4.4 mm (0–14 mm) with IBEF compared with 2.4 ± 3.7 mm (0–13 mm) without IBEF and 4.4 ± 6.1 mm (0–25 mm) regarding the preoperative measurement (Table 2). For cases without a surgery-related deficit at the three months follow-up, the mean distance with IBEF between the resection cavity and the fiber tracts was 3.1 ± 4.5 mm (0–14 mm) compared with a mean distance of 0.7 ± 1.2 mm (0–2 mm) for three cases with surgery-related deficits or postoperative deficits, which were lost to follow-up (*p* = 0.541; Figure 4). 

Including patients without language status follow-up at the three month examination, no permanent surgery-related language deficit occurred for any distance between the resection cavity and language eloquent fiber tracts greater than 2 mm. For a distance between the resection cavity and language fiber tracts ≤ 2 mm, the positive predictive value for permanent surgery-related deficits was 0.136.

The difference between the minimum distance between the resection cavity and eloquent fiber tracts for rigid fusion and IBEF was 1.0 ± 1.4 mm (0–5 mm) for motor eloquent fiber tracts, with a maximum range of 3 mm shift inward to 5 mm outward. In four selected cases, we used subcortical stimulation to verify the location of the CST. According to the rule that 1 mA corresponds to 1 mm, the localization of the CST as visualized by tractography correlated with subcortical stimulation in two cases before the application of IBEF, and in all cases after the application of IBEF. These data were published recently in detail [18]. For language eloquent fiber tracts, the difference was 1.0 ± 1.5 mm (0–6 mm), ranging from 3 mm inward to 6 mm outward. 

## 4. Discussion

### 4.1. Correlation of IBEF-Based Tractography and Clinical Outcome

This present study’s results confirm the clinical relevance of tractography as adjusted by IBEF in a large cohort and show its chances of providing accurate data. The mean ± standard deviation (range) distance after the application of IBEF was 5.0 ± 2.9 mm (0–10 mm) in patients without surgery-related motor deficits compared with 1.1 ± 1.6 mm (0–5 mm) in patients who showed new permanent surgery-related motor deficits postoperatively (*p* < 0.0001). The distance was 0.7 ± 1.2 mm (0–2 mm) in patients with new permanent language deficits compared with 3.1 ± 4.5 mm (0–14 mm) in patients without new permanent surgery-related language deficits (*p* = 0.541). This validates IBEF for its application during surgery as an addition to anatomical ioMRI data. The visualization of a corrected version of preoperative tractography on ioMRI imaging provides additional information relevant to the process of decision making regarding further resection under the goal of preserving functionality in cases where tumor residue is identified on the ioMRI scans.

### 4.2. IBEF-Based Tractography for Risk Stratification

In this study, a minimum distance >5 mm was identified as the limit required to avoid surgery-related deficits of motor function, and a distance >2 mm for surgery-related language deficits. These findings correlate with previous studies on preoperative risk assessment based on nTMS-based fiber tracking, showing no permanent surgery-related motor deficits for a distance between the CST and the lesion greater than 8 to 12 mm [23,24]. For permanent surgery-related language deficits, a cut-off value of 8 mm in preoperative nTMS-based FT was measured [25].

In this study, the minimum distance necessary to avoid surgery-related deficits of motor function was remarkably higher compared with language function. This might be due to a better compensation regarding cortical plasticity as well as a higher interindividual variability in language function [26,27,28]. A higher probability of aberrant fibers in nTMS-based language FT, due to a variable positive predictive value and different approaches of FT, has to be considered [28,29,30].

By acquiring further FT data on ioMRI, a risk score could be implemented, estimating the hazard of permanent or transient surgery-related deficits, based on the distance between eloquent fiber tracts and the resection cavity. Furthermore, precise intraoperative tractography might enhance the performance of subcortical stimulation. Providing accurate data regarding the distance between the resection cavity on ioMRI and eloquent FT at the planned site of further resection might help the surgeon to apply the correct stimulation intensities for direct subcortical stimulation.

### 4.3. Clinical Relevance of Intraoperative Brain Shift

Previous studies showed that brain shift is present in almost all cases of tumor resection, which also affects eloquent fiber tracts [31,32]. In order to compensate for intraoperative brain shift, neuronavigation needs intraoperative enhancement, such as 5-aminolevulinic acid, fluorescein, intraoperative ultrasound, and ioMRI [33,34]. In the present study, the minimum distance between the resection cavity and eloquent fiber tracts between rigid fusion and IBEF was 1.0 ± 1.4 mm (0–5 mm) for motor eloquent fiber tracts with a maximum range from 3 mm shift inward to 5 mm outward. For language eloquent fiber tracts, the difference was 1.0 ± 1.5 mm (0–6 mm), ranging from 3 mm inward to 6 mm outward. Regarding fiber tracts such as the CST, previous studies showed an outward shift of fiber tracts during resection in the majority of cases [35,36]. However, inward shifting of fiber tracts was reported in up to 50% [35,36], which increases the risk of surgery-related deficits compared with the risk assessment according to the spatial relationships in preoperative tractography.

Based on the results of the present study, which showed that mean differences between rigid and elastic fusion equaled the extent of brain shift of around 1 mm on a subcortical level, the severity of brain shift and its impairment of tractography were overestimated in the past, at least on a subcortical level. With this in mind, even preoperative tractography is highly accurate for motor and language tractography. This has already been shown by prior publications regarding the accuracy of uncorrected tractography as examined by direct subcortical stimulation [6,7,37,38].

### 4.4. Limitations

IBEF aims to adjust the preoperatively calculated tractography to the ioMRI scans. However, it is still an approximation and only serves as compensation for brain shift identified on the ioMRI scans. It may lack precision in the further course of surgery due to subsequent brain shift. Therefore, MEP monitoring is still needed to constantly provide accurate information during surgery. Furthermore, we analyzed the distance between the resection cavity and the fiber tracts as the only factor in relation to the postoperative patient outcome. Other factors including edema and ischemia were not considered in this approach.

## 5. Conclusions

IBEF enables adjustments to preoperatively acquired tractography by compensating for brain shift after ioMRI to provide precise data. Therefore, IBEF could be implemented into the intraoperative workflow. By providing additional information on eloquent fiber tracts after performing ioMRI, it might support further resection after ioMRI imaging by providing a precise risk assessment of surgery-related deficits in terms of further resection and aiding further resection by providing data for subcortical stimulation.

Still, even preoperative tractography for subcortical resection of gliomas is highly accurate for motor and language tractography. At least for this subcortical anatomy, the severity of brain shift was considerably overestimated in the past.

## Figures and Tables

**Table 1:** Distance between the motor eloquent fiber tracts and the intraoperative magnetic resonance imaging (MRI) resection cavity. This table shows the distance between the motor eloquent fiber tracts and the resection cavity depicted in the intraoperative MRI. Cases without surgery-related deficits at the 3 month follow-up (FU) (including patients with transient surgery-related deficits) are compared with cases with permanent surgery-related deficits at the 3 month FU (including patients with surgery-related deficits lost to FU). Distances are shown for measurements with IBEF, without IBEF, and preoperative measurements.

Motor Examination Three Months Postoperatively	Distance Fiber Tracts—Resection Cavity with IBEF (mm)	Distance Fiber Tracts—Resection Cavity without IBEF		Distance Fiber Tracts—Preoperative Tumor	
		Distance (mm)	*p*-Value (vs. IBEF)	Distance (mm)	*p*-Value (vs. IBEF)
All cases (mm)	3.6 ± 3.2 (0–10)	2.7 ± 3.0 (0–9)	0.001	3.2 ± 3.5 (0–15)	0.243
Transient or no surgery-related deficits (mm)	5.0 ± 2.9 (0–10)	3.7 ±3.0 (0–9)	0.001	4.3 ± 3.7 (0–15)	0.151
Permanent surgery-related deficits (mm)	1.1 ± 1.6 (0–5)	0.9 ± 2.1 (0–8)	0.750	1.3 ± 1.9 (0–6)	0.820
*p*-value (deficit/no deficit)	<0.001	0.001	-	0.001	-

**Table 2:** Distance between the language eloquent fiber tracts and the intraoperative MRI resection cavity. This table shows the distance between the language eloquent fiber tracts and the resection cavity depicted in the intraoperative MRI. Cases without surgery-related deficits at the 3 months follow-up (FU) (including patients with transient surgery-related deficits) are compared with cases with permanent surgery-related deficits at 3 months FU (including patients with surgery-related deficits lost to FU). Distances are shown for measurements with ioMRI-based elastic fusion (IBEF), without IBEF, and preoperative measurements.

Language Examination Three Months Postoperatively	Distance Fiber Tracts—Resection Cavity with IBEF	Distance Fiber Tracts—Resection Cavity without IBEF		Distance Fiber Tracts—Preoperative Tumor	
		Distance (mm)	*p*-Value (vs. IBEF)	Distance (mm)	*p*-VALUE (vs. IBEF)
All cases (mm)	2.9 ± 4.4 (0–14)	2.4 ± 3.7 (0–13)	0.180	4.4 ± 6.1 (0–25)	0.015
No surgery-related deficits (mm)	3.1 ± 4.5 (0–14)	2.6 ± 3.8 (0–13)	0.234	4.4 ± 6.4 (0–25)	0.042
Surgery-related deficits (mm)	0.7 ± 1.2 (0–2)	0.0 ± 0.0 (0–0)	>0.999	4.0 ± 3.6 (0–7)	0.500
*p*-value (deficit/no deficit)	0.541	0.284	-	0.825	-

## Data Availability

The data presented in this study are available in Table 1 and Table 2, Figure 3 and Figure 4, and Supplementary Tables S1 and S2.

## Supplementary Materials

The following are available online at https://www.mdpi.com/article/10.3390/cancers13081787/s1, Table S1: Patient characteristics for intraoperative MRI with motor tractography, Table S2: Patient characteristics for intraoperative MRI with language tractography.
