# Supplementary material for: Tractography for Subcortical Resection of Gliomas Is Highly Accurate for Motor and Language Function: ioMRI-Based Elastic Fusion Disproves the Severity of Brain Shift
Source: Cancers (Basel). 2021 Apr 9;13(8):1787. doi: 10.3390/cancers13081787 (PMC8068819; doi:10.3390/cancers13081787)
Supplement: Supplementary file 1 [file cancers-13-01787-s001.pdf]

cancers

Supplementary Materials

Tractography for Subcortical Resection of Gliomas is Highly Accurate for Motor and Language Function: ioMRI-Based Elastic Fusion Disproves the Severity of Brain Shift

Supplementary Table S1. Patient characteristics for intraoperative MRI with motor tractography

This table gives an overview of patient and tumor characteristics for 46 cases of patients obtaining intraoperative magnetic resonance imaging (MRI). Grading of motor deficits was conducted according to the British Medical Research Council (BMRC) scale.

Gender<br>(n (%))	Females<br>Males	15 (32.6%)<br>31 (67.4%)
Age at surgery<br>(mean and range)		52.1 (26.2 – 81.1) years
Primary tumor<br>(n (%))	WHO II°<br>WHO III°<br>WHO IV°	9 (19.6%)<br>14 (30.4%)<br>23 (50.0%)
Tumor-affected hemisphere<br>(n (%))	Right<br>Left	22 (47.8%)<br>24 (52.2%)
Extent of resection<br>(n (%))	Gross total resection<br>Subtotal resection	39 (84.8%)<br>7 (15.2%)
Resection after intraoperative MRI<br>(n (%))	Yes<br>No	13 (28.3%)<br>33 (71.7%)
Duration of intraoperative MRI<br>(mean and range)		65 (30-105) min
Preoperative motor deficits<br>(n (%))	BMRC 5/5<br>±4/5<br>≤3/5	33 (71.7%)<br>7 (15.2%)<br>6 (13.0%)
Motor deficits 5 days postoperatively<br>(n (%))	BMRC 5/5<br>±4/5<br>≤3/5	21 (45.7%)<br>5 (10.9%)<br>20 (43.5%)
Motor deficits 3 months	BMRC 5/5	16 (34.8%)

postoperatively<br>(n (%))	±4/5<br>≤3/5<br>Lost to follow-up	8 (17.4%)<br>8 (17.4%)<br>14 (30.4%)

Table S2: Patient characteristics for intraoperative MRI with language tractography
This table gives an overview of patient and tumor characteristics for the 32 cases with language tractography and intraoperative magnetic resonance imaging (MRI). Grading of language deficits was conducted according to the modified Aachen Aphasia Test (AAT).

Gender<br>(n (%))	Female<br>Male	20 (62.5%)<br>12 (37.5%)
Age at surgery<br>(mean and range)		52.1 (25.2 – 79.2) years
Primary tumor<br>(n (%))	WHO II°<br>WHO III°<br>WHO IV°	2 (6.3%)<br>12 (37.5%)<br>18 (56.3%)
Tumor-affected hemisphere<br>(n (%))	Right<br>Left<br>Both	0 (0.0%)<br>31 (96.9%)<br>1 (3.1%)
Extent of resection<br>(n (%))	GTR<br>STR	20 (62.5%)<br>12 (37.5%)
Resection after intraoperative MRI<br>(n (%))	Yes<br>No	8 (25.0%)<br>24 (75.0%)
Duration of intraoperative MRI<br>(mean and range)		64 (35-100) min
Preoperative motor deficits<br>(n (%))	Modified AAT<br>0<br>1<br>2<br>3	 21 (65.6%)<br>7 (21.9%)<br>3 (9.4%)<br>1 (3.1%)
Language deficits 5 days postoperatively<br>(n (%))	Modified AAT<br>0<br>1<br>2	 15 (46.9%)<br>9 (28.1%)<br>4 (12.5%)

	3	4 (12.5%)
Language deficits 3 months postoperatively (n (%))	Modified AAT<br>0<br>1<br>2<br>3<br>Lost to follow-up	 20 (62.5%)<br>7 (21.9%)<br>2 (6.3%)<br>2 (6.3%)<br>1 (3.1%)
